# Compound heterozygosity for Southeast Asian hereditary persistence of fetal hemoglobin and β^0^-thalassemia results in thalassemia intermedia: Pedigree analysis and genetic research in a family from South China. A case report

**DOI:** 10.1097/MD.0000000000037446

**Published:** 2024-03-08

**Authors:** Guangli Wang, Huiping Deng, Peng Peng, Haiqing Zheng, Baodong Tian, Chunjiang Zhu

**Affiliations:** a Genetics and Precision Medicine Laboratory, Affiliated Hospital of Guilin Medical University, Guilin, China; b Department of Neonatology, The Third People’s Hospital of Hubei province, Wuhan, China; c Department of Radiology, The First Affiliated Hospital of Guangxi Medical University, Nanning, China; d Department of Pediatrics, Affiliated Hospital of Guilin Medical University, Guilin, China.

**Keywords:** β-case report, genotype, iron overload, SEA-HPFH, thalassemia

## Abstract

**Rationale:**

Compound heterozygotes for deletional β-thalassemia can be difficult to diagnose due to its diverse clinical presentations and no routine screenings. This can lead to disease progression and delay in treatment.

**Patient concerns:**

We reported pedigree analysis and genetic research in a family with rare β-thalassemia.

**Diagnosis:**

Pedigree analysis and genetic research demonstrated that the patient was a compound heterozygote for β-thalassemia CD17/Southeast Asian hereditary persistence of fetal hemoglobin deletion, inherited from the parents. Magnetic resonance imaging T2* examination revealed severe iron deposition in the liver. Echocardiography revealed endocardial cushion defect.

**Interventions:**

The patient was treated with Deferasirox after receiving the final molecular genetic diagnosis. The initial once-daily dose of Deferasirox was 20 mg/kg/d.

**Outcomes:**

The patient discontinued the medication three months after the first visit. Two years later, the patient visited the Department of Hepatobiliary and Pancreatic Diseases. He was recommended to undergo splenectomy after surgical repair of the congenital heart disease. However, the patient refused surgical treatment because of the economic burden.

**Lessons:**

We report that fetal hemoglobin is a sensitive indicator for screening large deletions of the β-globin gene, which can be effectively confirmed by the multiplex ligation-dependent probe amplification assay. In non-transfusion-dependent thalassemia patients, iron status assessment should be regularly performed, and iron chelation treatment should be initiated early. This case will provide insights for the diagnosis of rare genotypes of β-thalassemia and has important implications for genetic counseling.

## 1. Introduction

A recent epidemiological survey indicated that the prevalence of β-thalassemia was approximately 6.66% in Guangxi, a province located in southwestern China.^[1]^ β-thalassemia is caused by either point mutations or large deletions in the β-globin (HBB) gene cluster.^[2]^ The most common HBB gene cluster deletions in the Chinese population are Southeast Asian hereditary persistence of fetal hemoglobin (SEA-HPFH) and Chinese ^G^γ^+^ (^A^γδβ)^0^-thalassemia. Heterozygotes of these deletions usually have no clinical manifestations or mildly reduced hemoglobin levels.^[3]^ However, when these deletional mutations are coinherited with heterozygous β-thalassemia, patients may present with thalassemia intermedia or thalassemia major.

Currently, commercial kits can detect the 17 most common point mutations of β-thalassemia, which accounts for approximately 90% of all mutation types in southern China. However, HBB gene cluster deletions are not included in routine screening.^[3]^ Compound heterozygotes for deletional β-thalassemia can be difficult to diagnose due to its diverse clinical presentations and no routine screenings. This can lead to disease progression and delay in treatment. Therefore, identification of rare HBB gene mutations plays a key role in the correct diagnosis and early treatment of patients with β-thalassemia.^[4]^

In this case, we analyzed correlations between genotype and phenotype in a patient with rare β-thalassemia, providing insight into the genetic counseling of patients with β-thalassemia and the prevention and control of this disease in Guangxi province.

## 2. Materials and methods

### 2.1. Clinical data

**Figure 1.**
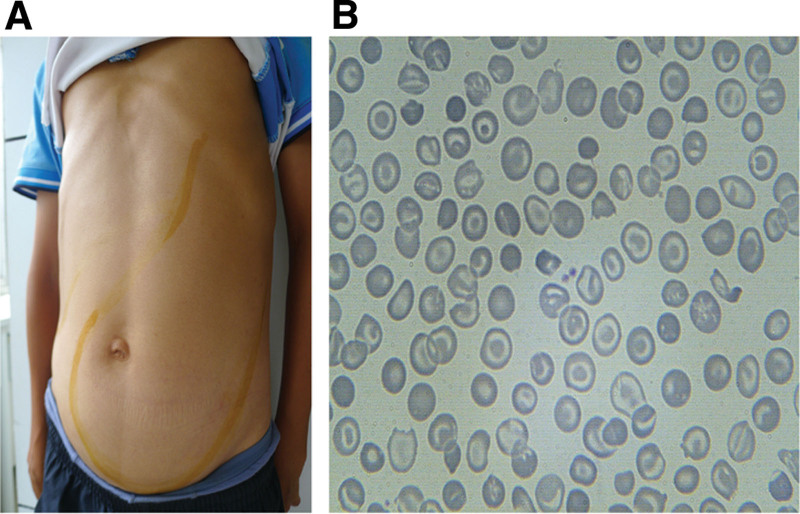
Physical signs and blood film from the proband suffering from thalassemia intermedia. (A) Abdomen enlargement caused by hepatomegaly and giant splenomegaly almost occupying the entire abdomen. (B) Peripheral blood smears showing microcytosis, hypochromia, increased target cells, and irregular contracted red blood cells.

A 32-year-old male proband was referred to the Affiliated Hospital of Guilin Medical University, Guilin, Guangxi Province, People’s Republic of China (PRC) for genetic counseling. He was admitted with a 30-year history of pallor and a 10-year history of fatigue and dizziness after activity. The patient had no history of blood transfusions. Physical examination revealed typical clinical characteristics of thalassemia intermedia pallor, jaundice, growth retardation, typical craniofacial changes, and abdominal enlargement caused by hepatomegaly and giant splenomegaly occupying almost the entire abdomen (Fig. 1A). He had a systolic murmur of grade 3/6 or more on the left edge of the sternum at the third or fourth rib. A routine blood examination revealed hypochromic microcytic anemia. Hemoglobin electrophoresis analysis showed high levels of hemoglobin A2 (HbA_2_; 4%) and fetal hemoglobin (HbF; 96%). Routine screening for thalassemia revealed that the proband was homozygous for the CD17 mutation, which was inconsistent with the clinical phenotype.

Pedigree and genetic analyses were performed on family members to establish a definite diagnosis. Pedigree members included the proband’s parents and elder sister. This study was approved by the Ethics Committee of the First Affiliated Hospital of Guilin Medical University. All participants were informed of the purpose of the study and provided written informed consent.

### 2.2. Research methods

#### 2.2.1. Hematological analysis.

Peripheral blood samples were collected from the proband and family members for hematological analysis. For morphological analysis, blood samples were collected from the proband and peripheral blood smears were stained using the Wright-Giemsa method. Serum ferritin (SF) levels were measured using enzyme-linked immunosorbent assay.

#### 2.2.2. Genetic analysis.

Peripheral blood was collected and genomic DNA was extracted using a genomic DNA extraction kit (Zhishan Biological, Xiamen, China). Gap polymerase chain reaction (gap-PCR; Yishengtang Biological, Shenzhen, China) and PCR reverse point hybridization (PCR-RDB; Yaneng Biological) were used to detect the common α-globin and HBB gene defects. Multiplex ligation-dependent probe amplification (MLPA) was used to detect HBB gene cluster deletions according to the manufacturer’s instructions (MRC-Holland, The Netherlands).^[3]^

#### 2.2.3. Imaging examination.

Iron content in the liver and heart of the proband was evaluated using magnetic resonance imaging (MRI).^[5]^ Iron values were calculated using T2* analysis spreadsheet software (CMR Tools, Essex, UK). The guidelines for MRI T2* measurements in iron overload for both the liver and heart are shown in Table 1.^[6]^ Echocardiography and abdominal ultrasound tests were performed in the proband using a Doppler ultrasonic apparatus (Philips, Best, The Netherlands).

**Table 1:** Guidelines for MRI T2* measurements in iron overload for both liver and heart.

Iron overload	Cardiac T2*, ms	Liver T2*, ms
None	>20	>6.3
Mild	14–20	2.6–6.3
Moderate	10–14	1.4–2.6
Severe*	<10	<1.4

## 3. Results

### 3.1. Abdominal ultrasound and echocardiography tests in the proband

**Figure 2.**
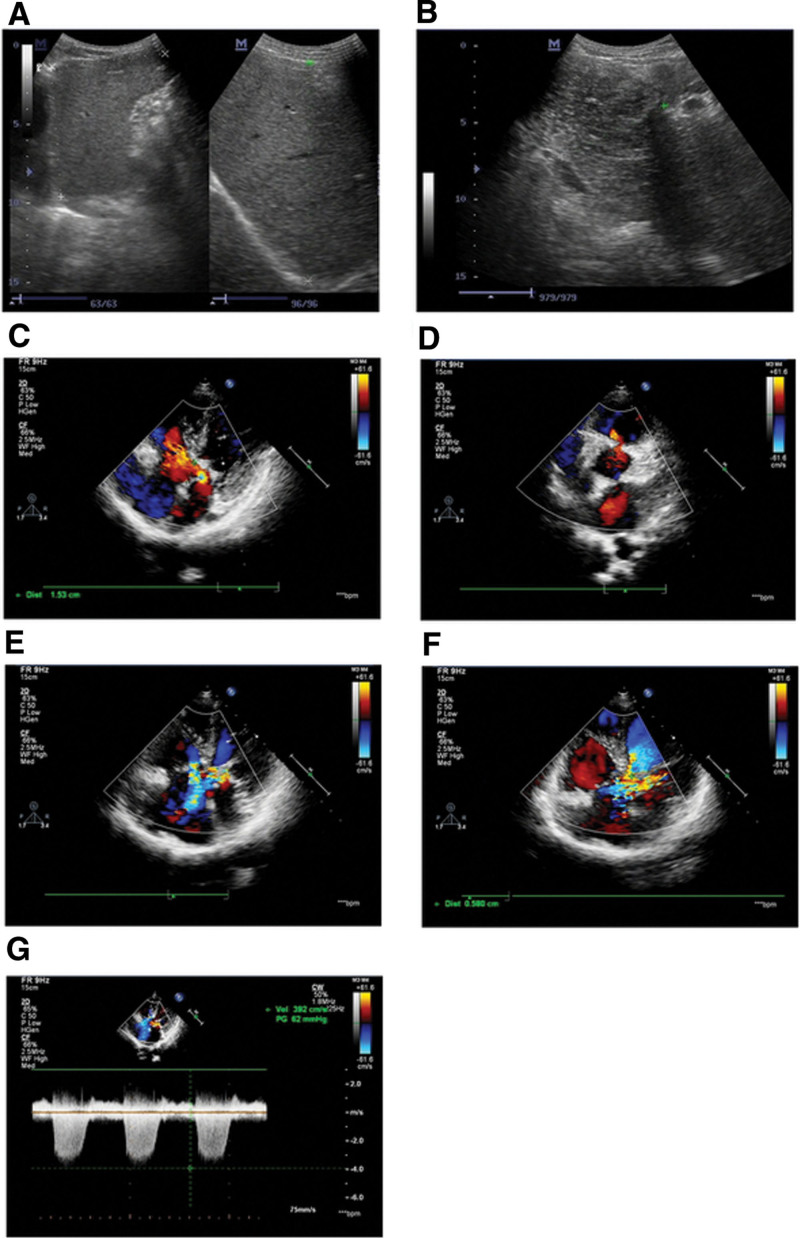
Proband ultrasonogram and echocardiography results. Ultrasonogram images show enlarged (A) liver and (B) spleen. (C–G) Echocardiography revealing endocardial cushion defect, including (C) atrial septal defect, (D) ventricular septal defect, (E) root interruption of anterior mitral valve, (F) echo interruption of tricuspid septal valve, and (G) pulmonary hypertension.

Abdominal ultrasonography showed that the patient’s liver was moderately enlarged, and its length under the costal margin was 4.7 cm. The spleen was markedly enlarged and occupied almost the entire abdomen (Fig. 2A and B).

The echocardiography results showed normal left ventricular function (LVEF 62.2%, LVFS 33.8%, LVES 3.46 cm, and LVED 5.23 cm) and pulmonary hypertension (pulmonary artery pressure 62 mm Hg) (Fig. 2C–G). In addition, echocardiography revealed atrial septal defect, ventricular septal defect, root rupture of the anterior mitral valve, and defect in the tricuspid septal valve, indicating an endocardial cushion defect, which is a type of congenital heart disease. Pulmonary hypertension is a frequent complication in patients with endocardial cushion defect.^[7]^ The patient presented with mild heart failure symptoms even with normal ejection fraction.

### 3.2. Myocardial and hepatic iron overload assessment in the proband

**Figure 3.**
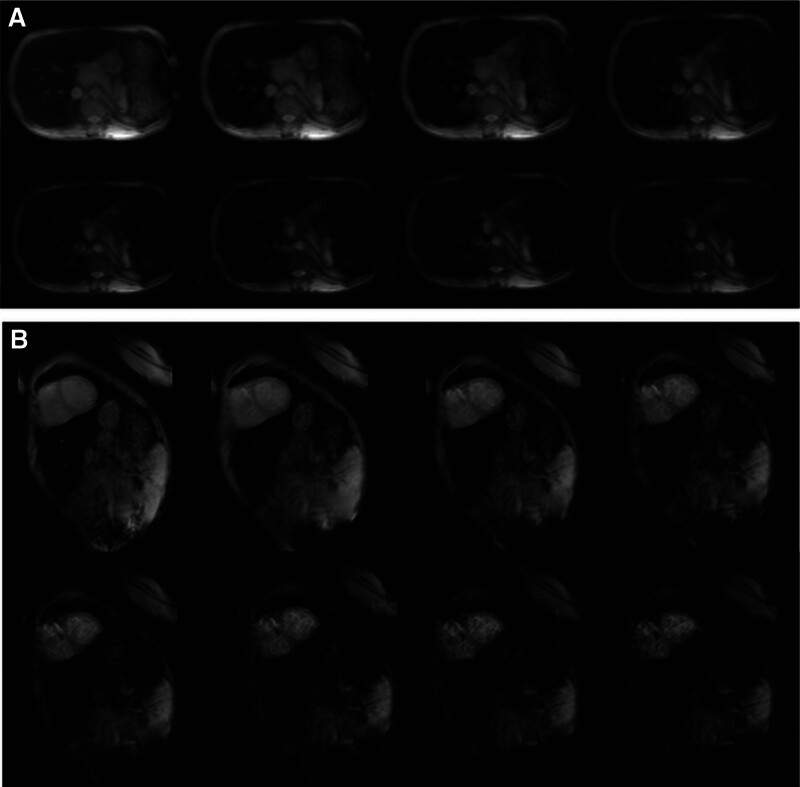
Magnetic resonance imaging (MRI) T2* measurements of (A) liver and (B) heart in the proband.

MRI T2* measurements were used to assess myocardial and hepatic iron overload in the patient (Fig. 3). Regarding the severity of liver iron concentration overload, the proband had a severe iron overload (T2* = 0.93 ms) and sharp decrease in signal intensity as the echo time increased. However, the myocardial iron concentration was normal (T2* = 27.7 ms).

### 3.3. Hematological parameters and red cell indices

Hematological analysis suggested hypochromic and microcytic anemia (Table 2). Hemoglobin electrophoresis revealed high levels of HbF (12%) in the father and normal HbF levels in the mother. SF levels in the proband were significantly elevated (2321 ng/mL). In addition, peripheral blood smears showed abnormal red blood cell morphology, including mild microcytosis, irregularly contracted cells, and increased target cells (Fig. 1B).

**Table 2:** Hematological and genotypic data of the family members.

No.	Relationship	Age	RBC (×10^12^)	Hb (g/L)	MCV (fL)	MCH (pg)	MCHC (g/L)	HbA (%)	HbA_2_ (%)	HbF (%)	Genotype
I-1	Father	80	4.23	92	68.6	18.2	266	83.6	4.4	12	αα/αα SEA-HPFH/β^N^
I-2	Mother	73	5.15	100	63.9	19.4	304	93.9	5.9	0.2	αα/αα β^17M^/β^N^
II-1	Proband	32	4.88	102	69.8	21.4	306	0	4.0	96	αα/αα SEA-HPFH/β^17M^
II-2	Sister	35	4.18	90	66.5	21.5	324	/	/	/	αα/αα β^17M^/β^N^

Hb = hemoglobin, HbA = hemoglobin A, HbA2 = hemoglobin A2, HbF = fetal hemoglobin, MCH = mean corpusonlar hemoglobin, MCHC = mean corpuscular hemoglobin con-centration, MCV = mean corpuscular volume, RBC = red blood cells.

### 3.4. Screening for common α-thalassemia and β-thalassemia mutations

**Figure 4.**
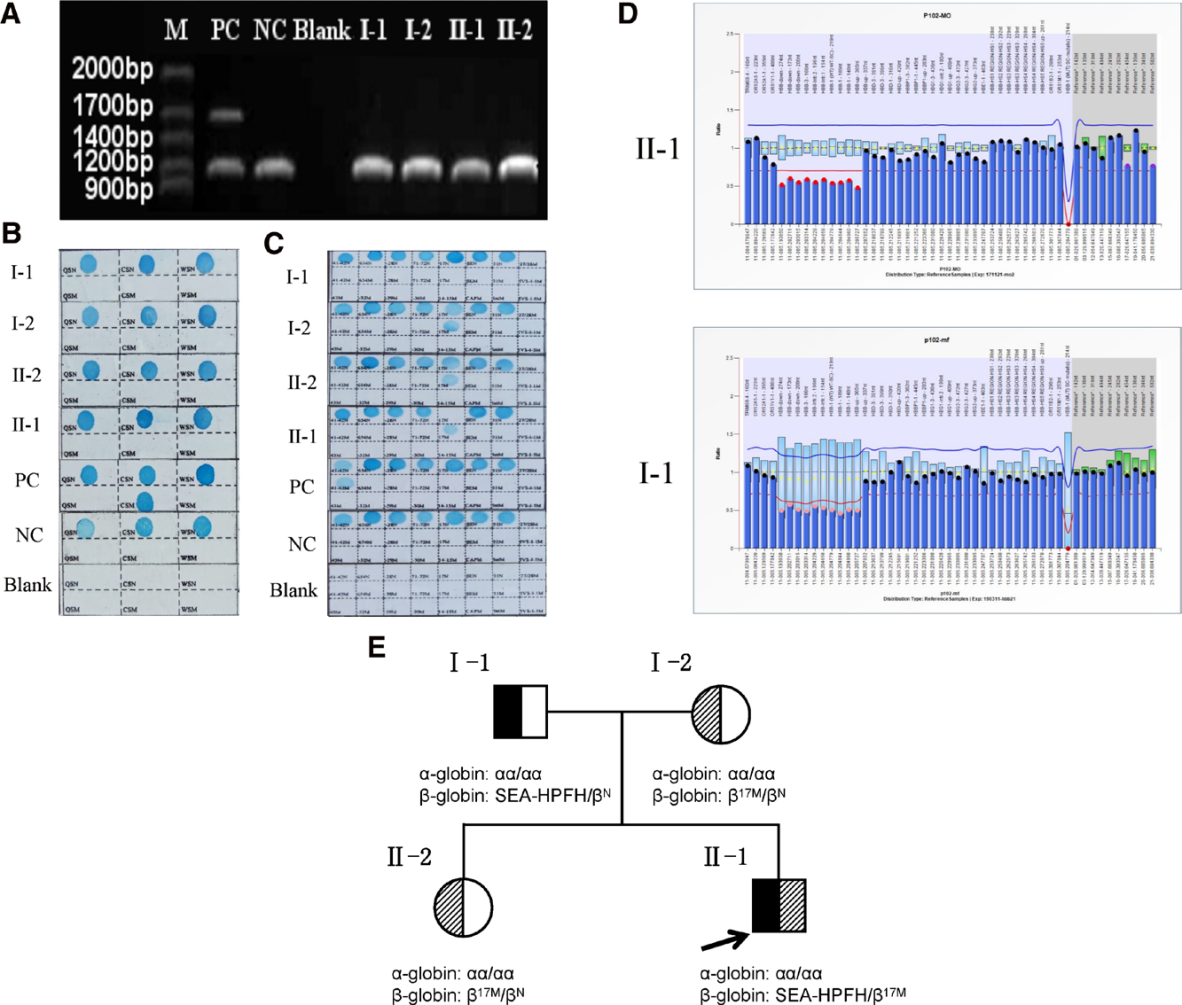
Common thalassemia genotype and multiplex ligation-dependent probe amplification (MLPA) analysis results of the family members. (A) Agarose electrophoresis showing the result of α gene deletion assay. (B) PCR reverse point hybridization (PCR-RDB) result of α gene mutation. (C) PCR-RDB result of β gene mutation. (D) MLPA analysis showing half dosages for ten probes located in the β-globin (HBB) gene in the proband and his father. Strips with red dots are probes with reduced ratio, dosage quotient range is between 0.4 and 0.65. When the detection probe of HBB point mutation (HBB: c. 20A > T, rs334) leading to sickle cell disease drops to 0, there is no corresponding mutation and therefore no detection signal generated in the samples. (E) The pedigree and summary of the molecular analysis in the family members. M = marker, NC = normal control, PC = positive control.

Gap-PCR combined with PCR-RDB (Fig. 4A–C) revealed that the proband was homozygous for the CD17 mutation. Family studies showed that the mother and elder sister were heterozygous for the CD17 mutation. Surprisingly, the father showed no mutation in the HBB gene. The hematological indices of the proband and father were not compatible with the β-thalassemia genotype. These results indicated that rare mutations in HBB may be present in the proband and father.

### 3.5. Detection of HBB gene deletion using MLPA

Genomic DNA from the proband and father was assessed for large fragment deletions in the HBB gene using MLPA analysis (Fig. 4D). The results indicated that the SEA-HPFH deletion was present in the proband and father.

### 3.6. Genetic analysis of the family

Molecular characterization revealed that the proband carried a CD17 mutation and SEA-HPFH deletion inherited from his parents. The father was heterozygous for SEA-HPFH deletion. The mother and elder sister were heterozygous for the CD17 mutation. The family pedigree is shown in Figure 4E.

### 3.7. Treatment and follow-up

**Figure 5.**
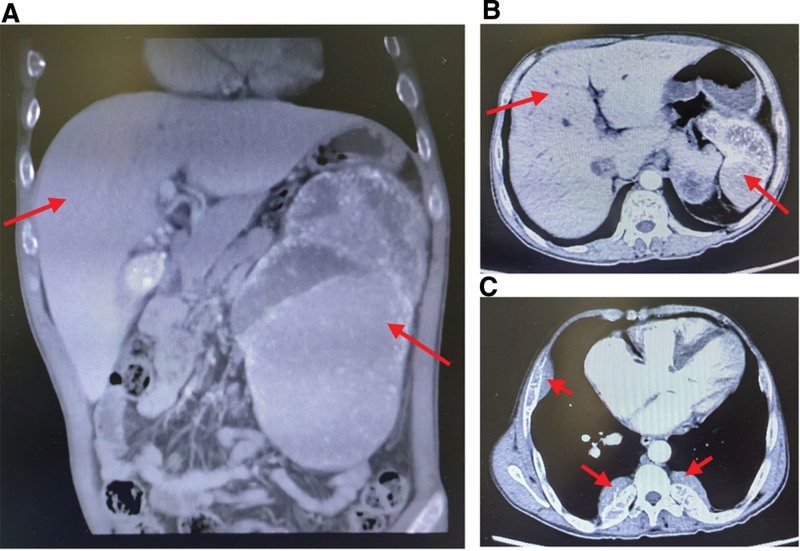
Follow-up computed tomography (CT) images 2 years after the first visit showing (A, B) hepatosplenomegaly and (C) extramedullary hematopoiesis in the ribs.

The patient was treated with Deferasirox after he received a final molecular genetic diagnosis of compound heterozygote for β-thalassemia SEA-HPFH/β^17M^. The initial once-daily dose of Deferasirox was 20 mg/kg/d. The patient discontinued the medication three months after the first visit. Two years later, he presented to the Department of Hepatobiliary and Pancreatic Diseases and due to upper abdominal pain for six months. Routine blood analysis revealed that the Hb level decreased to 92 g/L. SF level was elevated at 2108.38 ng/mL. Serum total bilirubin level increased to 52.8 µmol/L. Computed tomography (CT) suggested extramedullary hematopoiesis in the 8th–11th ribs (Fig. 5). The patient was recommended to undergo splenectomy after surgical repair of the congenital heart disease. However, the patient refused surgical treatment because of the high medical costs.

### 3.8. Review of the literature

To gain a thorough understanding of the clinical features of SEA-HPFH in the Chinese population, we performed an extensive literature review of publications deposited in the PubMed database. Seven literature records were retrieved from 2009 to 2022.^[3,4,8–12]^ In these studies, 2160 subjects with elevated HbF levels were screened for SEA-HPFH using Gap-PCR or MLPA. Among them, 204 (9.44%) subjects were diagnosed with SEA-HPFH thalassemia, including 184 SEA-HPFH carriers, 13 cases of SEA-HPFH combined with α-thalassemia, and 7 cases of SEA-HPFH combined with β-thalassemia. The SEA-HPFH combined with β-thalassemia cases included 3 cases of SEA-HPFH/β^41-42^, 2 cases of SEA-HPFH/β^IVS-II-654^, 1 case of SEA-HPFH/β^IVS II-180 (T > C)^, and 1 case of SEA-HPFH/β^-43(C > T)^. Patients with SEA-HPFH combined with α-thalassemia had no or mild anemia, with high HbA_2_ (2.8–5.3%) and HbF (11–26%) levels. Patients with SEA-HPFH combined with β-thalassemia presented with mild anemia, with elevated HbA_2_ (3.0–3.9%) and extremely high HbF (>95%) levels. The hematological and genetic diagnosis data of the 204 individuals with SEA-HPFH are shown in Table 3.

**Table 3:** Hematological and gene diagnosis data of 204 individuals with SEA-HPFH.

Cases	Hb (g/L)	MCV (fL)	MCH (pg)	HbA (%)	HbA_2_ (%)	HbF (%)	α-Genotype	β-Genotype	Ref.
proband	102	69.8	21.4	0	4.0	96	αα/αα	SEA-HPFH/β^17^	/
50	137.8 ± 14.0	76.6 ± 4.3	25.1 ± 1.5	74.2 ± 4.2	4.0 ± 0.6	21.8 ± 4.8	αα/αα	SEA-HPFH/β^N^	^[4]^
97	/	76.6 ± 5.86	25.2 ± 1.93	/	3.9 ± 1.00	21.53 ± 6.3	αα/αα	SEA-HPFH/β^N^	^[11]^
7	128.1 ± 7.2	76.96 ± 3.09	24.96 ± 1.17	74.96 ± 1.69	2.83 ± 0.20	22.21 ± 1.75	αα/αα	SEA-HPFH/β^N^	^[9]^
10	129.5 ± 21.3	77.5 ± 5.3	25.4 ± 1.3	74.8 ± 4.0	4.2 ± 0.6	21.0 ± 4.3	αα/αα	SEA-HPFH/β^N^	^[3]^
14	109 ± 15	77.19 ± 7.14	26.05 ± 2.33	73.19 ± 4.91	3.36 ± 0.77	23.45 ± 5.14	αα/αα	SEA-HPFH/β^N^	^[8]^
1	161	70.9	24.9	76.7	4.40	18.9	/	SEA-HPFH/β^N^	^[12]^
5	124 ± 5.8	76.98 ± 3.66	25 ± 1.25	/	4.42 ± 0.64	20.84 ± 5.53	αα/αα	SEA-HPFH/β^N^	^[10]^
1	/	68.50	27.90	/	4.70	15.00	-α^3.7^/αα	SEA-HPFH/β^N^	^[11]^
1	/	72.50	23.50	/	4.00	26.50	-α^4.2^/αα	SEA-HPFH/β^N^	^[11]^
3	130.0 ± 11.1	69.5 ± 0.6	22.2 ± 0.2	76.4 ± 4.1	3.7 ± 0.5	19.9 ± 4.1	--^SEA^/αα	SEA-HPFH/β^N^	^[4]^
7	/	72.4 ± 4.39	23.29 ± 1.09	/	3.98 ± 0.93	15.55 ± 6.95	--^SEA^/αα	SEA-HPFH/β^N^	^[11]^
1	119	75.9	23.8	74.2	4	21.8	αα^WS^/αα	SEA-HPFH/β^N^	^[3]^
1	/	73.30	21.10	/	3.20	/	αα/αα	SEA-HPFH/β^41-42^	^[11]^
1	104	63.0	22.0	0.5	3.8	95.7	αα/αα	SEA-HPFH/β^41-42^	^[9]^
1	104	63.4	20.7	0.5	3.8	95.7	αα/αα	SEA-HPFH/β^41-42^	^[10]^
1	112	64.7	22.4	0.9	3.9	95.2	αα/αα	SEA-HPFH/β^IVS-II-654^	^[9]^
1	113	63.7	21.0	1.3	3.0	95.7	αα/αα	SEA-HPFH/β^IVS-II-654^	^[10]^
1	/	75.40	24.40	/	/	/	αα/αα	SEA-HPFH/β^(IVS II-180 T > C)^	^[11]^
1	/	79.00	24.90	82.6	2.30	15.10	αα/αα	SEA-HPFH/β^-43(C > T)^	^[11]^

Hb = hemoglobin, HbA = hemoglobin A, HbA2 = hemoglobin A2, HbF = fetal hemoglobin, MCH = mean corpusonlar hemoglobin, MCV = mean corpuscular volume.

## 4. Discussion

β-thalassemia can be clinically categorized as β-thalassemia minor, intermedia, and major.^[2]^ Patients with thalassemia intermedia present with mild-to-moderate anemia (Hb 7–10 g/dL) and do not require regular blood transfusions.^[2,13]^ The proband in this case showed typical clinical manifestations of β-thalassemia intermedia. Iron overload is the leading cause of death and disability in patients with β-thalassemia intermedia.^[14,15]^ SF concentrations and the MRI T2* method are the clinical gold standard for assessing iron deposition. Iron chelation therapy is an effective treatment for preventing iron overload-related complications such as cirrhosis and heart failure. According to blood transfusion requirements, thalassemia can be phenotypically classified into transfusion-dependent and non-transfusion-dependent thalassemia (NTDT). Thalassemia international federation recommends that iron chelation therapy is given to NTDT patients with SF level ≥ 800 μg/L. In this case, the proband, with elevated SF level of 2321 μg/L, was non-transfusion dependent. Therefore, iron chelation treatment should be initiated as early as possible. Cardiac dysfunction is considered the primary cause of death in patients with thalassemia intermedia.^[14]^ Fortunately, the patient in this study showed no signs of iron deposition in the heart, although there was severe hepatic iron overload. Previous studies have concluded that elevated liver iron levels increase the risk of cardiac events.^[16,17]^ In contrast, several recent studies have demonstrated a less obvious correlation between liver iron concentration and heart iron.^[15,18]^ One possible reason for this disconnection may be an organ-specific mechanism of iron uptake and release. The liver is the natural storage organ for iron and is the most commonly involved organ in patients with iron overload, whereas cardiac involvement is relatively late.^[14]^ The different mechanisms for uptake of iron into the liver and heart result in different rate of organ iron loading.

β-thalassemia is caused by genetic variants in the HBB gene cluster, which results in the imbalance of α-globin and β-globin chains. More than 220 mutations, including point mutations and large fragment deletions, have been reported to be associated with β-thalassemia.^[2]^ Routine blood analysis in the proband showed mild hypochromic microcytic anemia with increased HbA_2_ level of 4.0% and HbF of 96.0%. Gap-PCR combined with PCR-RDB revealed that the patient was homozygous for CD17 mutation. Based on clinical experience and literature reports, CD17(A > T) mutation is one of the most frequent β-thalassemia mutations in the Chinese population. This mutation is a nonsense mutation which causes the complete absence of β-globin chain synthesis. Patients with the CD17 homozygous mutation present with thalassemia major.^[19]^ Therefore, the phenotype was inconsistent with the genotype observed in this case. The extremely high HbF level suggested that the patient may carry a deletional β-thalassemia.^[20]^ The molecular characterization showed that the proband carried the SEA-HPFH deletion, inherited from the father.

Deletions in the HBB gene cluster are distinctly uncommon.^[20]^ The molecular mechanism of SEA-HPFH is that the large 27 kb deletion in the HBB gene cluster leads to the reverse β- to γ-globin switch, which in turn results in the persistent high levels of HbF in the adult, compensating for the β-globin deficiency.^[21]^ HbF is a sensitive indicator for the screening of SEA-HPFH, and patients with elevated HbF need further examination for deletion mutations in the HBB gene cluster to make a definite diagnosis. SEA-HPFH typically causes mild microcytic hypochromic anemia. However, coinheritance with β-thalassemia is previously identified to result in thalassemia major or intermedia.^[22]^ Thanh et al^[21]^ reported a case of a compound heterozygote for β-thalassemia (IVS-II-654)/SEA-HPFH deletion, whose phenotype was β-thalassemia intermedia. In contrast, Cianetti et al^[23]^ reported a healthy case who had heterocellular HPFH interacting with both β^+^- and δβ-thalassemia. In this case, the patient had mild anemia and was independent from blood transfusion for in the past thirty years. A complex pattern of phenotypes may occur because of varying degrees of severity, which depends on the type of β-hemoglobin mutations involved. Although anemia was not evident, the patient in our study developed serious complications associated with chronic hemolysis and iron overload, including severe hepatomegaly and splenomegaly. In NTDT patients, iron overload results largely from increased intestinal absorption of iron, which is caused by ineffective erythropoiesis. Hepcidin, a hepatic peptide hormone, controls intestinal absorption, plasma concentrations, and tissue distribution of iron.^[24]^ Ineffective erythropoiesis plays a suppressive role in hepcidin expression. Systemic iron overload is largely due to low hepcidin levels and the consequent increased intestinal absorption of iron. In NTDT patients, iron overload usually becomes obvious in their 30s and 40s.^[15]^ Consistent with these results, severe iron deposition in the liver was observed in the proband. Many studies have indicated a strong correlation between iron overload and various morbidities in NTDT patients.^[25]^ Therefore, early detection and iron chelation treatment are needed; iron chelation treatment should be initiated as early as possible for the proband in this study.

## 5. Conclusion

Diagnosis of compound heterozygotes for SEA-HPFH and β-thalassemia can be missed due to its diverse clinical presentations and no routine screenings. Clinicians should pay close attention to patients with genotype–phenotype discordance. HbF is a sensitive indicator for screening of large deletions in the HBB gene, which can be effectively confirmed using the MLPA assay. In NTDT patients, iron status assessment should be regularly performed, and iron chelation treatment should be initiated early. These findings will provide ideas for the diagnosis of rare genotypes of β-thalassemia and have important implications for genetic counseling.

## Author contributions

**Conceptualization:** Guangli Wang, Chunjiang Zhu.

**Data curation:** Huiping Deng, Baodong Tian.

**Formal analysis:** Peng Peng.

**Funding acquisition:** Chunjiang Zhu.

**Investigation:** Huiping Deng, Haiqing Zheng.

**Methodology:** Peng Peng, Haiqing Zheng, Baodong Tian.

**Project administration:** Guangli Wang, Chunjiang Zhu.

**Software:** Peng Peng, Baodong Tian.

**Writing – original draft:** Guangli Wang, Huiping Deng.

**Writing – review & editing:** Chunjiang Zhu.
